# Consensus Control of Saturated Multi-Agent Systems with Heterogeneous Asymmetric Saturation Constraints Under Flexible Topologies

**DOI:** 10.3390/s26061923

**Published:** 2026-03-18

**Authors:** Zhanxiu Wang, Jikun Yang, Hao Yang, Zhenghong Jin

**Affiliations:** 1 School of Artificial Intelligence, Yancheng Teachers University, Yancheng 224002, China; zhanxiuwang@126.com (Z.W.); yangh01@yctu.edu.cn (H.Y.); 2 School of Electrical and Electronic Engineering, Nanyang Technological University, Singapore 639798, Singapore; 3 College of Information Engineering, Yancheng Institute of Technology, Yancheng 224051, China; 4 School of Mechanical and Aerospace Engineering, Nanyang Technological University, Singapore 639798, Singapore

**Keywords:** consensus control, saturated multi-agent systems, heterogeneous asymmetric constraints, directed switching topology

## Abstract

This paper investigates the consensus control of saturated continuous-time multi-agent systems with heterogeneous asymmetric saturation constraints under directed switching topologies of joint connectivity. Notably, the saturation level is asymmetric for each individual agent, and furthermore, these levels differ across the entire agent network. It is proven, by tools of the asymptotic stability and comparison principle, that conventional distributed protocols suffice to achieve consensus under the assumption of a uniformly quasi-strongly connected topology. This result fundamentally establishes the inherent tolerance of consensus algorithm to such generic, non-identical asymmetric saturation constraints. Numerical simulations are carried out to verify the effectiveness of the theoretical results.

## 1. Introduction

Driven by rapid advances in network technologies, computing power, and collaborative theory, multi-agent systems have garnered considerable attention from the system and control community over the past two decades. This sustained attention stems from a key advantage: the ability to solve complex problems through distributed coordination, thereby achieving superior scalability, flexibility, and robustness compared to single-agent systems. It is this advantage that has propelled the advancement of the multi-agent systems from a theoretical framework into practical application, leading to their widespread adoption across numerous domains including distributed optimization [1,2,3,4,5], formation control [6,7,8,9,10], consensus [11,12,13,14,15,16,17,18,19], and so forth.

As a fundamental collective behavior, consensus control in multi-agent systems has garnered sustained research interest. Consensus is defined as the process whereby agents, using local information, adjust their states until agreement is reached on a quantity of concern. Research in this area primarily concerns two key aspects: the agent dynamics and the information interaction topology among the agents. While early research in multi-agent systems concentrated on the topological conditions for consensus, the focus has since expanded to include increasingly complex agent dynamics, transitioning from strong, specific connectivity to weaker, more general topological assumptions. Specifically, using tools from algebraic graph theory and stochastic matrix theory, ref. [20] showed that consensus is achievable for linear first-order multi-agent systems with undirected and jointly connected communication topologies. As a natural extension, ref. [21] established that for single-integrator multi-agent systems under directed topologies, the necessary and sufficient condition for consensus is the existence of a spanning tree. Upon this basis, ref. [22] addressed the consensusability problem for linearly interconnected multi-agent systems over undirected graphs by demonstrating its equivalence to simultaneous stabilization and by providing linear-programming-based conditions, which reveal that weak physical coupling and densely connected graphs are favorable. With directed switching topologies, ref. [23] discussed the global target aggregation and state agreement problems for a group of continuous-time agents. However, these results are mainly focused on fixed or undirected switching topologies, while more realistic factors were not taken into account.

In multi-agent systems, input constraints that arise naturally from the physical limitations of actuators, communication bandwidth restrictions, and practical safety requirements are ubiquitous and pose a persistent challenge for cooperative control. This problem is particularly significant because such constraints can severely degrade system performance, trigger instability, or even preclude the achievement of consensus [11,24,25]. Thus, it is of practical significance to investigate how input saturation affects multi-agent consensus. In recent years, a considerable number of results have been published addressing the consensus control problem of multi-agent systems with input saturation constraints [11,16,24,26,27,28,29,30,31,32,33,34,35,36,37]. In particular, the authors of [35] considered the event-based consensus problem for linear heterogeneous multi-agent systems with symmetric input saturation, where the topology among agents is assumed to contain a directed spanning tree. Based on the developed finite-time consensus protocol under a fixed undirected connected topology, ref. [33] investigated a class of high-order multi-agent systems subject to symmetric bounded control inputs and external disturbances. Ref. [31] solved the fault-tolerant consensus problem for multi-agent systems with input constraints, where the interaction topology is switchable but must remain undirected and connected. Ref. [28] demonstrated that a prevalent linear consensus controller remains effective for first-order integrator systems with input saturation under general directed network topologies. Building on this, ref. [11] further investigated the consensus problem for multi-agent systems with heterogeneous asymmetric input saturation using only local state information, although the network topology, while allowed to be directed, was required to be strongly connected. In [24], the effect of input saturation with asymmetric levels was studied for multi-agent consensus, where the topology is allowed to switch but is required to be jointly strongly connected. Clearly, for switching topologies satisfying the uniformly quasi-strongly connected condition, the consensus problem for saturated continuous-time multi-agent systems with asymmetric constraints has yet to be solved.

In this paper, we investigate the consensus control problem for saturated continuous-time multi-agent systems under general directed and switching topologies. The topology is allowed to switch arbitrarily, provided that a uniformly quasi-strongly connected condition is satisfied. Moreover, unlike most existing works that assume homogeneous or symmetric input constraints [26,38,39,40], we consider the more general case of heterogeneous and asymmetric saturation constraints. To better illustrate the advancements achieved by our paper over previous results in consensus control of saturated multi-agent systems, a comparative analysis is provided in Table 1. The main contributions of this paper are as follows.

- This paper proposes a new method for solving the consensus control problem in saturated multi-agent systems subject to heterogeneous asymmetric saturation constraints. Furthermore, the effect of input saturation on consensus is investigated. By employing asymptotic stability theory and the comparison principle, we prove that consensus under conventional protocols is robust to arbitrary saturation levels.
- A sufficient and necessary condition on the saturation levels is established to ensure global asymptotic consensus. The proposed saturated controller is fully distributed, requiring neither knowledge of neighbors’ saturation levels nor of the global network topology, only the information of the relative state between agents.
- The network topology is allowed to be time-varying and may be disconnected at any time instant, provided that a uniformly quasi-strongly connected assumption is satisfied. Thereby, this paper extends the result of [11] from fixed to switching topologies and generalizes that of [24] from uniform joint strong connectivity to its quasi-strong counterpart, which enhances the flexibility of the network.

The rest of this paper is organized as follows. The consensus control problem formulation and the main results for saturated multi-agent systems with heterogeneous asymmetric saturation constraints are presented in Section 2. The convergence analysis of the main results is provided in Section 3. To verify the effectiveness of the theoretical results, two numerical examples are given in Section 4. Finally, some concluding remarks are drawn in Section 5.

**Notations and Definitions:** To facilitate the analysis, we first introduce some notations and definitions. The identity function on $R_{+}$ is denoted by Id, and the composition of two functions $\chi_{1},\chi_{2}:R_{+}\to R_{+}$ is written as $\chi_{1}\circ \chi_{2}$. We call a function $\alpha :R_{+}\to R_{+}$ a class K function if it is positive definite, continuous, and strictly increasing. Similarly, a function $\beta :R_{+}\times R_{+}\to R_{+}$ is classified as KL if it satisfies: (i) for each fixed t≥0, the function β(·,t) is of class K; (ii) for each fixed s≥0, the function β(s,·) is decreasing and satisfies $\lim_{t\to \infty }\beta \left(s,t\right)=0$. For the purpose of our discussion, we also introduce new function classes. We call a function $\beta :R_{+}\times R_{+}\to R_{+}$ a class ${\mathrm{IL}}^{+}$ function if it satisfies: (i) β is of class KL and satisfies β(s,0)=0 for s≥0; (ii) for any specific T>0, there exist continuous, positive definite, and non-decreasing functions $\alpha_{1},\alpha_{2}$ less than Id, such that for all $s\in R_{+}$, $\beta \left(s,t\right)\geq \alpha_{1}\left(s\right)$ for t∈[0,T] and $\beta \left(s,t\right)\leq \alpha_{2}\left(s\right)$ for t∈[T,∞). It should be noted that any ${\mathrm{IL}}^{+}$ is a KL function. We call a function $\beta :R\times R_{+}\to R$ a class IL function if there exist $\beta^{\prime },\beta^{\prime \prime }\in {\mathrm{IL}}^{+}$, such that for t≥0, $\beta \left(s,t\right)=-\beta^{\prime }\left(-s,t\right)$ for s<0 and $\beta \left(s,t\right)=\beta^{\prime \prime }\left(s,t\right)$ for s≥0. These new functions are used to precisely characterize the transient and asymptotic behavior of the states of the controlled agents.

The network topology is described by a digraph G=(N,E(t)), with the vertex set N corresponding to the agents and the edge set E(t)⊆N×N representing the information interaction at time *t*. An edge (j,i)∈E if and only if that agent *i* can access the state information of agent *j*. For convenience of discussion, the following notions will be introduced to model the agent interaction topology. A digraph G is said to be quasi-strongly connected (QSC) if there exists a node c∈N, referred to as the center, such that for every node i∈N, there is a directed path from *c* to *i*. For a switching digraph G, the union digraph over a time interval $\left[t_{a},t_{b}\right)$ as $G\left(\left[t_{a},t_{b}\right)\right)=\left(N,{⋃}_{t\in [t_{a},t_{b})}E\left(t\right)\right)$. A switching digraph G is said to be uniformly quasi-strongly connected (UQSC) when there is a positive constant *T*, such that G([t,t+T)) is QSC, and this holds for all t≥0. A switching digraph G(t) has an edge dwell time $\tau_{D}>0$ if for any t∈[0,∞) and any directed edge (j,i)∈E(t), there exists a $t^{\prime }\geq 0$ depending on *t*, such that $t\in [t^{\prime },t^{\prime }+\tau_{D})$ and (j,i)∈E(ς) for $\varsigma \in [t^{\prime },t^{\prime }+\tau_{D})$.

## 2. Problem Statement and Main Results

Consider the consensus control of multi-agent systems composed of *N* agents, and the dynamics of agent i∈N:={1,2,…,N} takes the following form:

(1) $${\dot{x}}_{i}={\mathrm{sat}}_{i}\left(\mu_{i}\left(t\right)\right)$$

for t≥0, where $x_{i},\mu_{i}\in R$ represent the state and the input of agent *i*, respectively, and sat(·) is a saturation function defined by

(2) $$\begin{array}{c} {\mathrm{sat}}_{i}\left(r\right)=\left\{\begin{array}{c} s_{i}^{L},\;\;\;\;\;\;\;\;\;\;\;\mathrm{for}\;r<s_{i}^{L},\\ r,\;\;\;\;\;\;\;\;\;\;\;\;\;\mathrm{for}\;s_{i}^{L}\leq r<s_{i}^{U},\\ s_{i}^{U},\;\;\;\;\;\;\;\;\;\;\;\mathrm{for}\;r\geq s_{i}^{U}, \end{array}\right. \end{array}$$

where $s_{i}^{L}\leq 0$ and $s_{i}^{U}\geq 0$ are unknown constants representing the lower and upper saturation bounds, respectively. Note that for each agent *i*, the condition $s_{i}^{U}=-s_{i}^{L}$ is not required. In addition, it is unnecessary to require that $s_{i}^{L}=s_{j}^{L}$ and $s_{i}^{U}=s_{j}^{U}$ for i,j∈N.

In this paper, the following assumption is made on the connectivity of the communication digraph.

**Assumption** **1.** *The switching digraph G(t)=(N,E(t)) is UQSC for all t≥0 with a positive time constant T and a positive edge dwell time $\tau_{D}$.*

**Remark** **1.** *Assumption 1 guarantees a lower bound on the duration of each edge, which is a standard assumption in switching systems that prevents digraphs from switching infinitely fast. Physically, once a communication link is established, it must persist for a strictly positive minimum duration to ensure information can be transformed from one agent to another.*

**Definition** **1.** *The multi-agent system* (1) *is said to reach global asymptotic consensus if there exists a function β∈KL, such that for arbitrary initial states of the agents, the property*

(3) $$\begin{array}{c} \max_{i,j\in N}|x_{i}\left(t\right)-x_{j}\left(t\right)|\leq \beta (\max_{i,j\in N}|x_{i}\left(0\right)-x_{j}\left(0\right)\left|,t\right) \end{array}$$

*holds for all t≥0.*

Consider the classical distributed protocol from [21]

(4) $$\begin{array}{c} \mu_{i}\left(t\right)=\sum_{j\in N_{i}\left(t\right)}\left(x_{j}\left(t\right)-x_{i}\left(t\right)\right). \end{array}$$

Combining this protocol with system (1) yields the following closed-loop dynamics

(5) $$\begin{array}{c} {\dot{x}}_{i}={\mathrm{sat}}_{i}\left(\sum_{j\in N_{i}\left(t\right)}\left(x_{j}\left(t\right)-x_{i}\left(t\right)\right)\right). \end{array}$$

Next, we employ a theorem to establish that the closed-loop saturated multi-agent system (5) achieves consensus under a condition of joint connectivity topology.

**Theorem** **1.** *Consider the controlled multi-agent system defined by* (5) *and* (2) *with $s_{i}^{L}\leq 0$ and $s_{i}^{U}\geq 0$. Under Assumption 1, global asymptotic consensus is achievable if and only if there does not exist an agent i such that $s_{i}^{L}=0$ or $s_{i}^{U}=0$.*

**Corollary** **1.** *Consider the controlled multi-agent system defined by* (5) *and* (2) *with $s_{i}^{L}\leq 0$ and $s_{i}^{L}=-s_{i}^{U}$. Under Assumption 1, global asymptotic consensus is achievable if and only if there does not exist an agent i such that $s_{i}^{L}=0$.*

**Remark** **2.** *Actually, Theorem 1 also means that global asymptotic consensus holds if and only if $s_{i}^{L}<0$ and $s_{i}^{U}>0$ for all i∈N.*

**Remark** **3.** *By establishing a fully distributed control protocol along with a necessary and sufficient condition on the saturation levels, Theorem 1 ensures robustness to heterogeneous asymmetric saturation constraints.*

## 3. Proof of the Main Results

In this section, we prove the main results for multi-agent systems under directed and time-varying topologies. To this end, we first introduce some key properties of the controlled agents in Section 3.1. Based on these properties, we then provide the proof of necessity and sufficiency for Theorem 1 in Section 3.2.

### 3.1. Necessary Propositions

Before given important propositions, we first introduce a technical lemma on convergence properties of a class of nonlinear systems.

**Lemma** **1** ([41])**.** *Consider the following initial value problem*

(6) $$\begin{array}{c} \dot{\zeta }=\alpha \left(\zeta \right),\;\;\;\zeta \left(0\right)=\zeta_{0} \end{array}$$

*where ζ∈R is the state, and α is non-increasing, locally Lipschitz, and satisfies α(0)=0, sα(s)<0 for all s≠0. There exists β∈IL such that for any $\zeta_{0}\in R$, it holds that*

(7) $$\begin{array}{c} \zeta \left(t\right)\leq \beta \left(\zeta_{0},t\right) \end{array}$$

*for t≥0.*

For convenience of discussion, we rewrite system (1) as

(8) $$\begin{array}{c} {\dot{x}}_{i}={\mathrm{sat}}_{i}\left(-N_{i}\left(x_{i}\left(t\right)-\eta_{i}\left(t\right)\right)\right). \end{array}$$

with $N_{i}$ being the neighbor number of agent *i* and

(9) $$\begin{array}{c} \eta_{i}\left(t\right)=\frac{\sum_{j\in N_{i}\left(t\right)}x_{j}\left(t\right)}{N_{i}}. \end{array}$$

representing the neighbor information that agent *i* can access in coordination control.

By treating $\eta_{i}$ as the external input to agent *i*, Proposition 1 provides estimates for both the upper and lower bounds of $x_{i}\left(t\right)$ and examines how $\eta_{i}$ affects these dynamics.

**Proposition** **1.** *For i∈N, consider system* (8) *with ${\mathrm{sat}}_{i}$ defined by* (2)*, where $s_{i}^{L}<0$ and $s_{i}^{U}>0$ are unknown constants. Then, there exist functions $\beta_{1i},\beta_{2i}\in \mathrm{IL}$ such that for any specific ${\underline{\eta }}_{i}\leq {\bar{\eta }}_{i}$, if $\eta_{i}\left(t\right)\in \left[{\underline{\eta }}_{i},{\bar{\eta }}_{i}\right]$ for $t\in [t_{0},T]$ with $T>t_{0}\geq 0$, then*

(10) $$\begin{array}{cc} -\beta_{1i}\left({\underline{\eta }}_{i}-x_{i}\left(t_{0}\right),t-t_{0}\right)+{\underline{\eta }}_{i} & \leq x_{i}\left(t\right)\\ & \leq \beta_{2i}\left(x_{i}\left(t_{0}\right)-{\bar{\eta }}_{i},t-t_{0}\right)+{\bar{\eta }}_{i} \end{array}$$

*for all $t\in [t_{0},T]$ and any initial state $x_{i}\left(t_{0}\right)\in R$.*

**Proof.**  By symmetry, it suffices to prove the second inequality in (10), as the first one follows analogously.

By Lemma 4.3 of [42], for any positive definite, non-decreasing function $\gamma_{i}$, there exist positive definite, non-decreasing functions ${\bar{\gamma }}_{i},{\underline{\gamma }}_{i}$, such that ${\underline{\gamma }}_{i}\left(s\right)\leq \gamma_{i}\left(s\right)\leq {\bar{\gamma }}_{i}\left(s\right)$ for all s≥0. By (8) and the definition of saturation function ${\mathrm{sat}}_{i}$, one sees that there is a non-increasing, locally Lipschitz function $\alpha_{i}$ satisfying $\alpha_{i}\left(0\right)=0$ and $s\alpha_{i}\left(s\right)<0$ for all s≠0, such that

(11) $$\begin{array}{cc} {\dot{x}}_{i}\left(t\right)= & {\mathrm{sat}}_{i}\left(-N_{i}\left(x_{i}\left(t\right)-\eta_{i}\left(t\right)\right)\right)\\ \leq & \alpha_{i}\left(x_{i}\left(t\right)-\eta_{i}\left(t\right)\right)\\ \leq & \alpha_{i}\left(x_{i}\left(t\right)-{\bar{\eta }}_{i}\right) \end{array}$$

for $t\in [t_{0},T]$, where the non-increasing property of $\alpha_{i}$ is used for the second inequality. Since ${\mathrm{sat}}_{i}$ is affine in $\left[S_{i}^{L},S_{i}^{U}\right]$ and constant outside, $\alpha_{i}$ is globally Lipschitz with constant $N_{i}$.

Define $\xi_{i}\left(t\right)$ as the solution to the following system

(12) $$\begin{array}{c} {\dot{\xi }}_{i}\left(t\right)=\alpha_{i}\left(\xi_{i}\left(t\right)-{\bar{\eta }}_{i}\right),\;\xi_{i}\left(t_{0}\right)=x_{i}\left(t_{0}\right). \end{array}$$

Then, an application of the comparison principle from [42] implies that

(13) $$\begin{array}{c} x_{i}\left(t\right)\leq \xi_{i}\left(t\right) \end{array}$$

holds on the interval $t\in [t_{0},T]$

Also note that $\alpha_{i}$ is locally Lipschitz and satisfies $\alpha_{i}\left(0\right)=0$ and $s\alpha_{i}\left(s\right)<0$ for all s≠0. By defining ${\tilde{\xi }}_{i}=\xi_{i}-{\bar{\eta }}_{i}$ and using (12), one sees that

(14) $$\begin{array}{c} {\dot{\tilde{\xi }}}_{i}\left(t\right)=\alpha_{i}\left({\tilde{\xi }}_{i}\left(t\right)\right). \end{array}$$

This together with Lemma 1 yields a class IL function $\beta_{2i}$ such that

(15) $$\begin{array}{c} {\tilde{\xi }}_{i}\left(t\right)\leq \beta_{2i}\left({\tilde{\xi }}_{i}\left(t_{0}\right),t-t_{0}\right),\;t\in \left[t_{0},T\right]. \end{array}$$

Recall that $x_{i}\left(t_{0}\right)=\xi_{i}\left(t_{0}\right)$. Then, it follows that

(16) $$\begin{array}{c} x_{i}\left(t\right)\leq \xi_{i}\left(t\right)\leq \beta_{2i}\left(x_{i}\left(t_{0}\right)-{\bar{\eta }}_{i},t-t_{0}\right)+{\bar{\eta }}_{i},\;\;t\in \left[t_{0},T\right]. \end{array}$$

This ends the proof of Proposition 1. □

We now consider the interconnected multi-agent system. For convenience, define the minimum and maximum states of the agents as

(17) $$\begin{array}{cc} \; & \underline{x}\left(t\right)=\min_{i\in N}x_{i}\left(t\right), \end{array}$$

(18) $$\begin{array}{cc} \; & \bar{x}\left(t\right)=\max_{i\in N}x_{i}\left(t\right). \end{array}$$

With these definitions, we present the following proposition.

**Proposition** **2.** *For $t\geq t_{0}\geq 0$, it holds that*

(19) $$\begin{array}{cc} \; & D^{+}\underline{x}\left(t\right)\geq 0, \end{array}$$

(20) $$\begin{array}{cc} \; & D^{+}\bar{x}\left(t\right)\leq 0. \end{array}$$

**Proof.**  We prove only (19) and omit the proof of (20), as it is analogous.

Define $L\left(t\right)=\{i\in N|x_{i}\left(t\right)=\underline{x}\left(t\right)\}$, which is the set of all agents achieving the minimum state value at time *t*. Then, by (8) and the definition of $\eta_{i}$ given by (9), one sees that

(21) $$\begin{array}{cc} D^{+}\underline{x}\left(t\right)= & \min_{i\in L(t)}{\dot{x}}_{i}\left(t\right)\\ = & \min_{i\in L(t)}{\mathrm{sat}}_{i}\left(-N_{i}\left(x_{i}\left(t\right)-\eta_{i}\left(t\right)\right)\right)\\ \geq & 0 \end{array}$$

with $D^{+}$ representing the Dini derivative ([43]). Here, the first inequality follows from the fact that $\underline{x}\left(t\right)-\eta_{i}\left(t\right)\leq 0$ and ${\mathrm{sat}}_{i}\left(s\right)\geq 0$ for s≥0. This completes the proof of Proposition 2.  ☐

### 3.2. Proof of Theorem 1

The proof of Theorem 1 consists of two parts: proving sufficiency and proving necessity. These two parts are addressed separately.

#### 3.2.1. Sufficiency

The sufficiency proof essentially shows that the maximum state difference between agents converges asymptotically. To this end, we first define some time scales and intervals. Let

(22) $$\begin{array}{c} \;\;\;\;{\bar{T}}_{k}=\left[kT^{*},\left(k+1\right)T^{*}\right), \end{array}$$

(23) $$\begin{array}{c} \;\;\;\;\;\;\;\;{\bar{T}}_{kl}=\left[kT^{*}+\left(l-1\right)T^{\prime },kT^{*}+lT^{\prime }\right), \end{array}$$

(24) $$\begin{array}{c} \;\;\;\;\;\;\;T_{ko}=\left[kT^{*}+NT^{\prime },\left(k+1\right)T^{*}\right), \end{array}$$

(25) $$\begin{array}{c} \;T^{*}=NT^{\prime }+\tau_{D}, \end{array}$$

(26) $$\begin{array}{c} T^{\prime }=T+2\tau_{D}. \end{array}$$

Then, a straightforward verification shows that ${\bar{T}}_{k}=T_{ko}\cup {⋃}_{l=1,\ldots ,N}{\bar{T}}_{kl}$. By partitioning the interval ${\bar{T}}_{k}$ into smaller subintervals ${\bar{T}}_{kl}$ and $T_{ko}$, we aim to investigate, step by step, how the state bounds evolve from agent to agent.

The remainder of the proof analyzes the motion of each controlled agent over an arbitrary interval ${\bar{T}}_{k}$ with $k\in Z_{+}$.

For each l=1,2,…,N, the index $i_{l}$ is recursively selected such that $i_{l}\in \left\{1,\ldots ,N\right\}\setminus \left\{i_{1},\ldots ,i_{l-1}\right\}$ and there exists an $i_{l^{\prime }}\in \left\{i_{1},\ldots ,i_{l-1}\right\}$ for which $\left(i_{l^{\prime }},i_{l}\right)\in E\left({\bar{T}}_{kl}\right)$. This recursive selection is feasible for all $i_{l}$ with l=1,2,…,N due to Assumption 1.

Let $N=P\cup P^{\prime }$ with

(27) $$\begin{array}{cc} \; & P=\left\{i\in N|x_{i}\left(kT^{*}\right)\leq \frac{\underline{x}\left(kT^{*}\right)+\bar{x}\left(kT^{*}\right)}{2}\right\}, \end{array}$$

(28) $$\begin{array}{cc} \; & P^{\prime }=\left\{i\in N|x_{i}\left(kT^{*}\right)>\frac{\underline{x}\left(kT^{*}\right)+\bar{x}\left(kT^{*}\right)}{2}\right\}. \end{array}$$

By the definition, both P and $P^{\prime }$ must contain at least one element.

(I) We first study the evolution of $x_{i_{1}}\left(t\right)$ for $t\in {\bar{T}}_{k}$ with $k\in Z_{+}$. Without loss of generality, we assume that $i_{1}\in P$. Following a similar line of reasoning, one can prove the case $i_{1}\in P^{\prime }$.

According to Proposition 2, it holds that $x_{i}\left(t\right)\leq \bar{x}\left(kT^{*}\right)$ for $t\in {\bar{T}}_{k}$. From the definition of $\eta_{i}$ in (9), it follows that $\eta_{i_{1}}\left(t\right)\leq \bar{x}\left(kT^{*}\right)$ for $t\in {\bar{T}}_{k}$. Then, by using Proposition 1, there exists a function $\beta_{2i_{1}}\in \mathrm{IL}$ such that for $t\in {\bar{T}}_{k}$

(29) $$\begin{array}{cc} x_{i_{1}}\left(t\right)\leq & \beta_{2i_{1}}\left(x_{i_{1}}\left(kT^{*}\right)-{\bar{\eta }}_{i_{1}},t-kT^{*}\right)+{\bar{\eta }}_{i_{1}}\\ \leq & \beta_{2i_{1}}\left(\frac{\underline{x}\left(kT^{*}\right)-\bar{x}\left(kT^{*}\right)}{2},T^{*}\right)+\bar{x}\left(kT^{*}\right)\\ = & -\beta_{2i_{1}}\left(\frac{\bar{x}\left(kT^{*}\right)-\underline{x}\left(kT^{*}\right)}{2},T^{*}\right)+\bar{x}\left(kT^{*}\right)\\ = & :-\alpha_{i_{1}}\left(\bar{x}\left(kT^{*}\right)-\underline{x}\left(kT^{*}\right)\right)+\bar{x}\left(kT^{*}\right). \end{array}$$

Here, the first inequality follows from the non-decreasing property of $\beta_{2i_{1}}\left(s,t\right)$ for fixed t≥0. It can be directly checked that $\alpha_{i_{1}}$ is continuous, positive definite, and satisfies $\alpha_{i_{1}}<\mathrm{Id}$.

(II) Next, we study the evolution of $x_{i_{j}}\left(t\right)$ for j=2,3,…,N during the interval $t\in [kT^{*}+jT^{\prime },\left(k+1\right)T^{*}]$ with $k\in Z_{+}$.

Consider the motion of $x_{i_{2}}$ during $t\in \left[t_{i_{2}},t_{i_{2}}+\tau_{D}\right]\subseteq {\bar{T}}_{k2}$. During this interval, it holds that

(30) $$\begin{array}{cc} \eta_{i_{2}}\left(t\right)= & \frac{\sum_{j\in N_{i_{2}}\left(t\right)\setminus \left\{i_{l^{\prime }}\right\}}x_{j}\left(t\right)+x_{i_{l^{\prime }}}\left(t\right)}{N_{i_{2}}}\\ \leq & \frac{\sum_{j\in N_{i_{2}}\left(t\right)\setminus \left\{i_{l^{\prime }}\right\}}\bar{x}\left(kT^{*}\right)-\alpha_{i_{1}}\left(\bar{x}\left(kT^{*}\right)-\underline{x}\left(kT^{*}\right)\right)+\bar{x}\left(kT^{*}\right)}{N_{i_{2}}}\\ = & -\alpha_{i_{2}}^{\prime }\left(\bar{x}\left(kT^{*}\right)-\underline{x}\left(kT^{*}\right)\right)+\bar{x}\left(kT^{*}\right) \end{array}$$

with $\alpha_{i_{2}}^{\prime }\left(s\right)=\alpha_{i_{1}}\left(s\right)/N_{i_{2}}$. This together with Proposition 1 yields a function $\beta_{2i_{2}}\in \mathrm{IL}$, such that

(31) $$\begin{array}{cc} x_{i_{2}}\left(t_{i_{2}}+\tau_{D}\right)\leq & \beta_{2i_{2}}\left(x_{i_{2}}\left(t_{i_{2}}\right)-{\bar{\eta }}_{i_{2}},\tau_{D}\right)+{\bar{\eta }}_{i_{2}}\\ \leq & \beta_{2i_{2}}\left(\alpha_{i_{2}}^{\prime }\left(\bar{x}\left(kT^{*}\right)-\underline{x}\left(kT^{*}\right)\right),\tau_{D}\right)+\bar{x}\left(kT^{*}\right)\\ & +\alpha_{i_{2}}^{\prime }\left(\bar{x}\left(kT^{*}\right)-\underline{x}\left(kT^{*}\right)\right)\\ = & :{\bar{\alpha }}_{i_{2}}\circ \alpha_{i_{2}}^{\prime }\left(\bar{x}\left(kT^{*}\right)-\underline{x}\left(kT^{*}\right)\right)+\bar{x}\left(kT^{*}\right)\\ & -\alpha_{i_{2}}^{\prime }\left(\bar{x}\left(kT^{*}\right)-\underline{x}\left(kT^{*}\right)\right)\\ = & -\left(\mathrm{Id}-{\bar{\alpha }}_{i_{2}}\right)\circ \alpha_{i_{2}}^{\prime }\left(\bar{x}\left(kT^{*}\right)-\underline{x}\left(kT^{*}\right)\right)+\bar{x}\left(kT^{*}\right)\\ = & :-{\tilde{\alpha }}_{i_{2}}\left(\bar{x}\left(kT^{*}\right)-\underline{x}\left(kT^{*}\right)\right)+\bar{x}\left(kT^{*}\right) \end{array}$$

for $t\in [t_{i_{2}},t_{i_{2}}+\tau_{D}]$. It can be seen that ${\tilde{\alpha }}_{i_{2}}$ is continuous, positive definite, and satisfies ${\tilde{\alpha }}_{i_{2}}<\mathrm{Id}$.

For the time interval $t\in [t_{i_{2}}+\tau_{D},\left(k+1\right)T^{*}]$, by the definition of $\eta_{i_{2}}$, one sees that

(32) $$\begin{array}{c} \eta_{i_{2}}\left(t\right)\leq \bar{x}\left(kT^{*}\right). \end{array}$$

Again, by using Proposition 1, one sees that there exists a $\beta_{2i_{2}}^{\prime }\in \mathrm{IL}$ such that

(33) $$\begin{array}{cc} x_{i_{2}}\left(t\right)\leq & \beta_{2i_{2}}\left(x_{i_{2}}\left(t_{i_{2}}+\tau_{D}\right)-{\bar{\eta }}_{i_{2}},t-t_{i_{2}}-\tau_{D}\right)+{\bar{\eta }}_{i_{2}}\\ \leq & \beta_{2i_{2}}\left(-{\tilde{\alpha }}_{i_{2}}\left(\bar{x}\left(kT^{*}\right)-\underline{x}\left(kT^{*}\right)\right),t-t_{i_{2}}-\tau_{D}\right)+\bar{x}\left(kT^{*}\right)\\ \leq & \beta_{2i_{2}}\left(-{\tilde{\alpha }}_{i_{2}}\left(\bar{x}\left(kT^{*}\right)-\underline{x}\left(kT^{*}\right)\right),T^{*}\right)+\bar{x}\left(kT^{*}\right)\\ = & :-\alpha_{i_{2}}\left(\bar{x}\left(kT^{*}\right)-\underline{x}\left(kT^{*}\right)\right)+\bar{x}\left(kT^{*}\right) \end{array}$$

for $t\in [t_{i_{2}}+\tau_{D},\left(k+1\right)T^{*}]$, where the non-decreasing properties of $\beta_{2i_{2}}\left(s,t\right)$ for fixed t≥0 and of $\beta_{2i_{2}}\left(-s,t\right)$ for fixed s>0 are used for the first and second inequalities, respectively. It can be verified that $\alpha_{i_{2}}$ is continuous, positive definite, and satisfies $\alpha_{i_{2}}<\mathrm{Id}$.

By recursively examining the cases of $x_{i_{j}}\left(t\right)$ for each j=3,…,N during $t\in [kT^{*}+jT^{\prime },\left(k+1\right)T^{*}]$ with $k\in Z_{+}$, we can prove that

(34) $$\begin{array}{c} x_{i_{2}}\left(t\right)\leq -\alpha_{i_{j}}\left(\bar{x}\left(kT^{*}\right)-\underline{x}\left(kT^{*}\right)\right)+\bar{x}\left(kT^{*}\right) \end{array}$$

with $\alpha_{i_{j}}$ being continuous, positive definite, and satisfying $\alpha_{i_{j}}<\mathrm{Id}$. Consequently, we can prove that for all i∈N

(35) $$\begin{array}{c} x_{i}\left(\left(k+1\right)T^{*}\right)\leq -\min_{i\in N}\alpha_{i}\left(\bar{x}\left(kT^{*}\right)-\underline{x}\left(kT^{*}\right)\right)+\bar{x}\left(kT^{*}\right). \end{array}$$

By defining

(36) $$\begin{array}{ccccccccc} & & & & & & & & \bar{x}\left(\left(k+1\right)T^{*}\right)=-\alpha \left(\bar{x}\left(kT^{*}\right)-\underline{x}\left(kT^{*}\right)\right)+\bar{x}\left(kT^{*}\right), \end{array}$$

(37) $$\begin{array}{cc} \; & \underline{x}\left(\left(k+1\right)T^{*}\right)=\underline{x}\left(kT^{*}\right) \end{array}$$

with $\alpha \left(s\right)=\min_{i\in N}\alpha_{i}\left(s\right)$ for all s≥0 and i∈N, we obtain

(38) $$\begin{array}{c} \tilde{x}\left(\left(k+1\right)T^{*}\right)\leq \tilde{x}\left(kT^{*}\right)-\alpha \left(\tilde{x}\left(kT^{*}\right)\right) \end{array}$$

for $k\in Z_{+}$ with $\tilde{x}\left(s\right)=\bar{x}\left(s\right)-\underline{x}\left(s\right)$ for s∈R. It then follows that there exists a β∈KL such that

(39) $$\begin{array}{c} \tilde{x}\left(kT^{*}\right)\leq \beta \left(\tilde{x}\left(0\right),kT^{*}\right). \end{array}$$

Then, $\tilde{x}\left(kT^{*}\right)\to 0$ as k→∞. Clearly, global asymptotic consensus is achieved. This completes the sufficiency proof of Theorem 1.

#### 3.2.2. Necessity

To prove necessity, we proceed by contradiction and we only need to construct a multi-agent system satisfying Assumption 1, such that if $s_{i}^{L}=0$ or $s_{i}^{U}=0$ holds for some i∈N, then global asymptotic consensus cannot be achieved.

We consider the case $s_{2}^{L}=0$. The case where $s_{2}^{U}=0$ can be treated similarly.

Construct a topology that is UQSC and satisfies the following conditions:

(i) Agent 1 is the leader, *i.e.*, there does not exists an edge (i,1)∈E;
(ii) For all t≥0, the edge (1,2) exists;
(iii) Agent 1 is the only neighbor of agent 2.

We randomly set the initial states of agents 3,4,…,N, and set the initial states of agents 2 and 1 such that $x_{2}\left(0\right)-x_{1}\left(0\right)\geq 0$. Then, by the system dynamics (5) and the definition of ${\mathrm{sat}}_{2}$, one sees that

(40) $$\begin{array}{c} {\dot{x}}_{2}\left(t\right)=0 \end{array}$$

for all t≥0. Then, one sees that

(41) $$\begin{array}{c} x_{2}\left(t\right)=x_{2}\left(0\right),\;\;\forall \;t>0. \end{array}$$

Since the state of agent 1 remains unchanged, then it holds that

(42) $$\begin{array}{c} x_{2}\left(t\right)-x_{1}\left(t\right)=x_{2}\left(0\right)-x_{1}\left(0\right),\;\;\forall \;t>0. \end{array}$$

Then, no matter how the other agents’ states vary, the state difference between agents 2 and 1 remains unchanged. Clearly, global asymptotic consensus cannot be achieved. By studying the other case, a similar conclusion can be obtained. This completes the necessity proof of Theorem 1.

## 4. Numerical Examples

To validate the effectiveness of the proposed control strategy in a sensor network context, two numerical examples are presented in this section. Both examples model a distributed sensor network comprising eight mobile sensing agents, indexed by the set N={1,2,3,4,5,6,7,8}. The first example demonstrates the practical effectiveness of the saturated control protocol and its robustness when actuator/sensor systems are subject to realistic physical limits. Specifically, we examine its performance under two types of input constraints: identical symmetric and non-identical asymmetric saturation levels. The second example is used to verify the necessity of the condition stated in Theorem 1.

**Figure 1:**
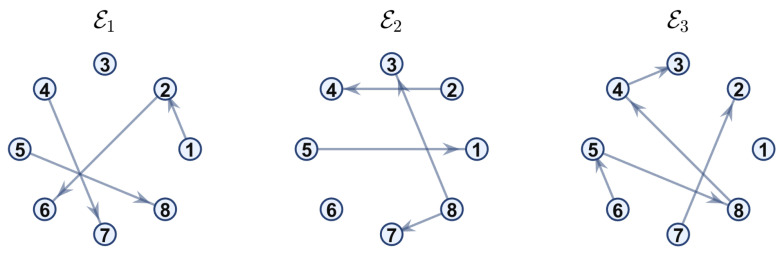
Digraphs used to represent the switching topology in Example 1.

**Example 1.** In this example, the communication network topology switches every 0.5 s among three digraphs, $G_{1}$, $G_{2}$, and $G_{3}$, with their edge sets shown in Figure 1.

**Case 1.** In this case, the closed-loop multi-agent system is designed as the form (8) with saturation function ${\mathrm{sat}}_{i}$ defined by

(43) $$\begin{array}{c} {\mathrm{sat}}_{i}\left(r\right)=\left\{\begin{array}{c} -5,\;\;\;\;\;\;\mathrm{for}\;r<-5,\\ r,\;\;\;\;\;\;\;\;\;\mathrm{for}\;-5\leq r<5,\\ 5,\;\;\;\;\;\;\;\;\mathrm{for}\;r\geq 5 \end{array}\right. \end{array}$$

for i=1,2,…,8. It can be verified that every saturation function ${\mathrm{sat}}_{i}$ is symmetric and that the agents share uniform saturation levels.

**Figure 2:**
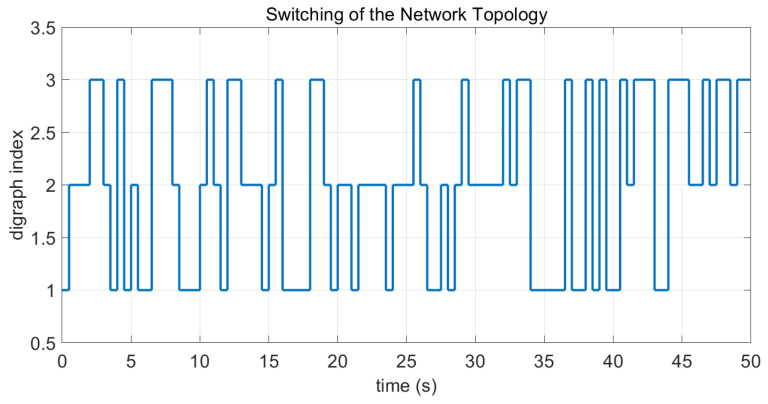
Switching sequence of the digraphs in Case 1 of Example 1.

**Figure 3:**
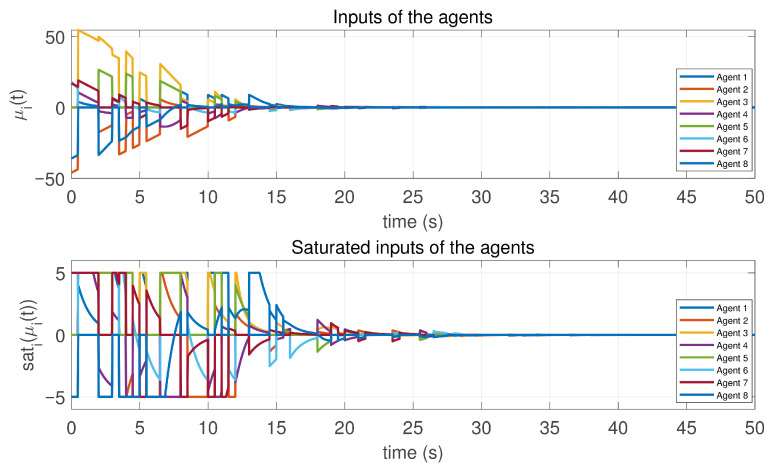
Inputs and saturated inputs of the controlled agents in Case 1 of Example 1.

**Figure 4:**
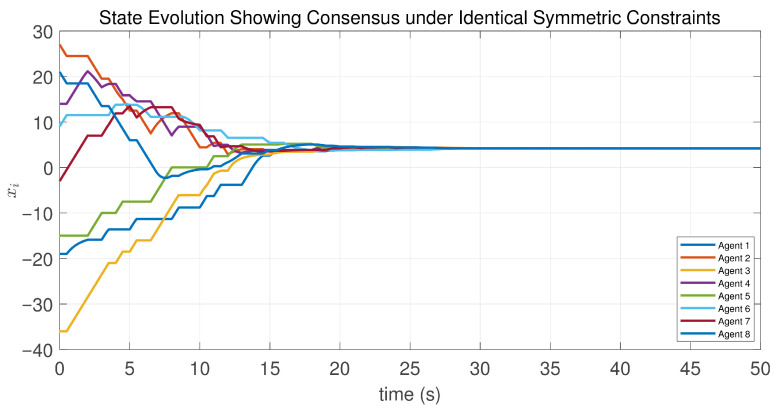
State evolution of the controlled agents in Case 1 of Example 1.

**Figure 5:**
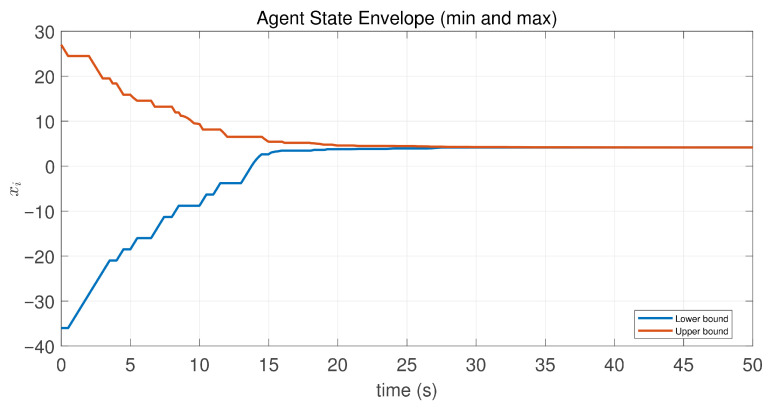
State envelopeof the controlled agents in Case 1 of Example 1.

**Figure 6:**
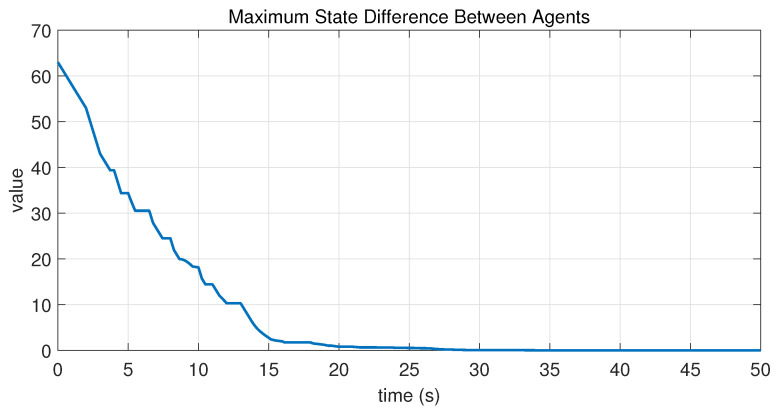
State evolution of $\max_{i,j\in N}\left|x_{i}-x_{j}\right|$ in Case 1 of Example 1.

In this case, the switching sequence of digraphs is shown in Figure 2. Because the union of the digraphs $G_{1}$ and $G_{2}$, the union of the digraphs $G_{1}$ and $G_{3}$, and the union of the digraphs $G_{2}$ and $G_{3}$ always contain a directed spanning tree, it can be seen that this topology satisfies the uniformly quasi-strongly connected assumption. With arbitrary initial conditions $x_{1}\left(0\right)=-19$, $x_{2}\left(0\right)=27$, $x_{3}\left(0\right)=-36$, $x_{4}\left(0\right)=14$, $x_{5}\left(0\right)=-15$, $x_{6}\left(0\right)=9$, $x_{7}\left(0\right)=-3$, and $x_{8}\left(0\right)=21$, the original and saturated control inputs of the agents are depicted in Figure 3. It can be seen that each input remains within its specified saturation bounds. The state convergence results are presented in Figure 4, Figure 5 and Figure 6, where the saturation constraints are identical and symmetric for all agents. The results demonstrate that the agents converge toward each other, with the maximum and minimum states asymptotically approaching a common value and the maximum state difference between agents asymptotically tending to zero. Clearly, the asymptotic consensus is achieved.

**Case 2.** In this case, the saturation function ${\mathrm{sat}}_{i}$ is defined by

(44) $$\begin{array}{c} {\mathrm{sat}}_{i}\left(r\right)=\left\{\begin{array}{c} -6-0.5i,\;\;\;\;\mathrm{for}\;r<-6-0.5i,\\ r,\;\;\;\;\;\;\;\;\;\;\;\;\;\;\;\mathrm{for}\;-6-0.5i\leq r<3+0.5i,\\ 3+0.5i,\;\;\;\;\;\;\mathrm{for}\;r\geq 3+0.5i \end{array}\right. \end{array}$$

for i=1,2,…,8. One can verify that each saturation function ${\mathrm{sat}}_{i}$ is asymmetric, and furthermore, the saturation levels differ among agents.

**Figure 7:**
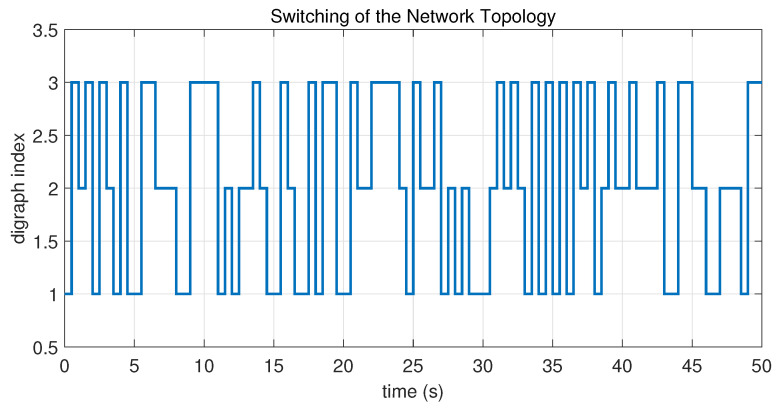
Switching sequence of the digraphs in Case 2 of Example 1.

**Figure 8:**
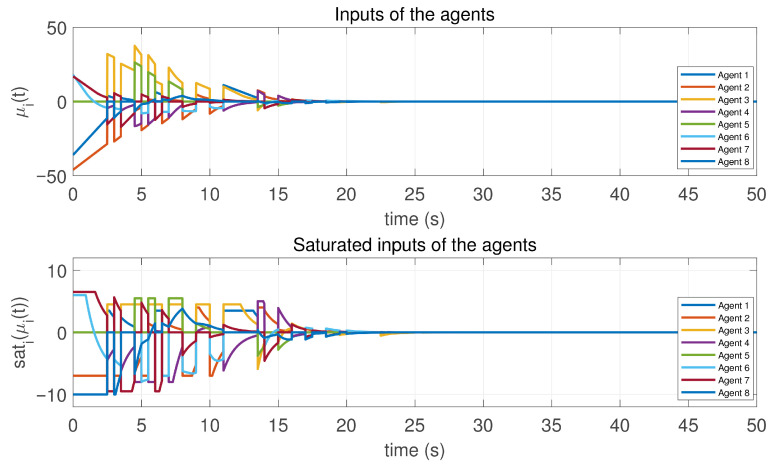
Inputs and saturated inputs of the controlled agents in Case 2 of Example 1.

**Figure 9:**
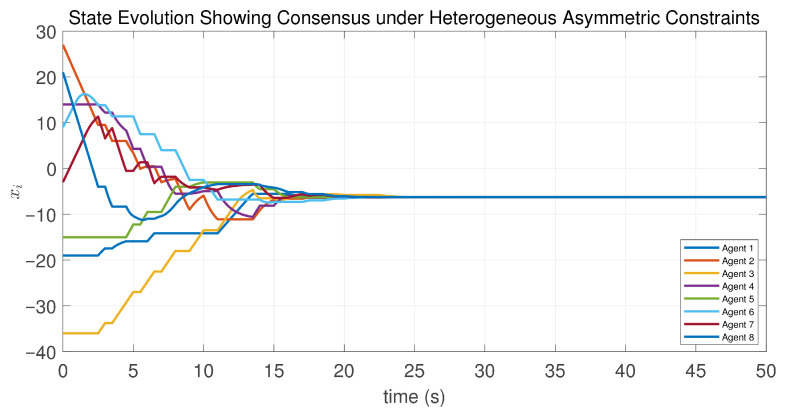
State evolution of the controlled agents in Case 2 of Example 1.

**Figure 10:**
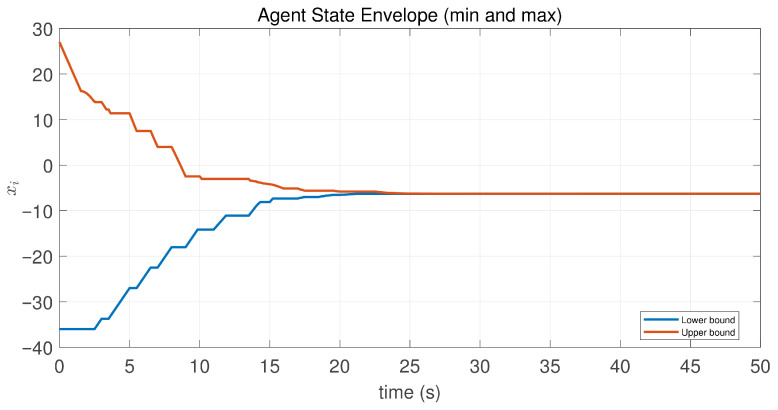
State envelope of the controlled agents in Case 2 of Example 1.

**Figure 11:**
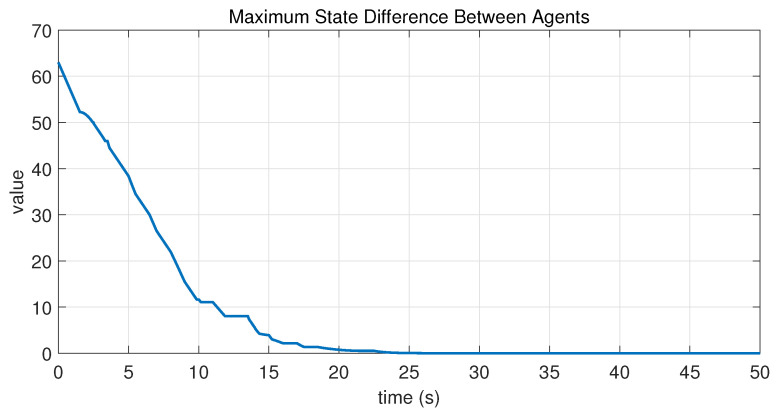
State evolution of $\max_{i,j\in N}\left|x_{i}-x_{j}\right|$ in Case 2 of Example 1.

In this case, the switching sequence of digraphs is shown in Figure 7. It can be shown that the topology thereby satisfies the connectivity requirement of Assumption 1. Under the same initial conditions as Case 1, the simulation results are presented in Figure 8, Figure 9, Figure 10 and Figure 11. As shown in Figure 8, even with heterogeneous saturation levels across agents, the control inputs (both original and saturated) are confined to the expected ranges. In addition, from Figure 9, Figure 10 and Figure 11, it can be observed that although the saturation constraints are heterogeneous and asymmetric, all agents converge to each other and asymptotic consensus is still guaranteed. This shows the tolerance of the considered consensus algorithm to non-identical asymmetric constraints, which is consistent with Theorem 1.

**Figure 12:**
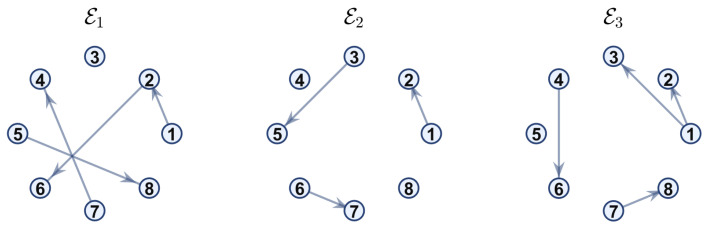
Digraphs used to represent the switching topology in Example 2.

**Example 2.** Similar to Example 1, the communication network topology also switches every 0.5 s among three digraphs, $G_{1}$, $G_{2}$, and $G_{3}$, with their edge sets shown in Figure 12. It should be noted that the edge (1,2) is contained in all digraphs.

**Figure 13:**
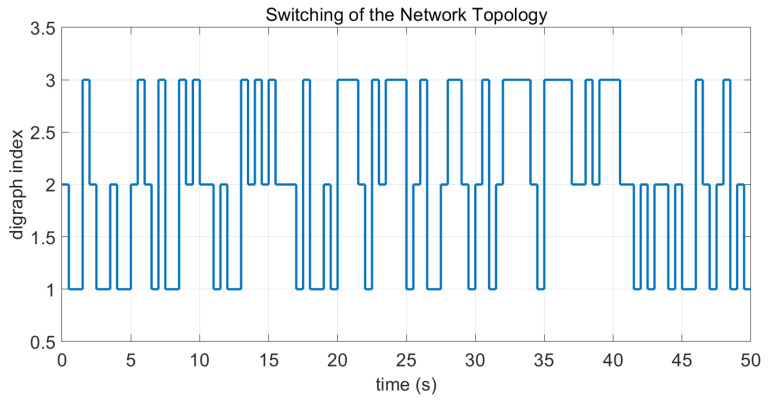
Switching sequence of the digraphs in Case 1 of Example 2.

**Case 1.** In this case, the switching sequence of the three digraphs is shown in Figure 13. It is not hard to see that the topology satisfies the connectivity assumption. We use the following saturation functions

(45) $$\begin{array}{c} {\mathrm{sat}}_{2}\left(r\right)=\left\{\begin{array}{c} 0,\;\;\;\;\;\;\;\;\mathrm{for}\;r<0,\\ r,\;\;\;\;\;\;\;\;\mathrm{for}\;0\leq r<4,\\ 4,\;\;\;\;\;\;\;\;\mathrm{for}\;r\geq 4 \end{array}\right. \end{array}$$

and

(46) $$\begin{array}{c} {\mathrm{sat}}_{i}\left(r\right)=\left\{\begin{array}{c} -6-0.5i,\;\;\;\;\mathrm{for}\;r<-6-0.5i,\\ r,\;\;\;\;\;\;\;\;\;\;\;\;\;\;\;\mathrm{for}\;-6-0.5i\leq r<3+0.5i,\\ 3+0.5i,\;\;\;\;\;\;\mathrm{for}\;r\geq 3+0.5i \end{array}\right. \end{array}$$

for i=1,3,4,5,6,7,8. It can be checked that $s_{2}^{L}=0$. Also, note that agent 1 is the only neighbor of agent 2. Then, one sees that

(47) $$\begin{array}{c} {\dot{x}}_{2}=0 \end{array}$$

when $x_{2}\left(t\right)-x_{1}\left(t\right)\geq 0$.

**Figure 14:**
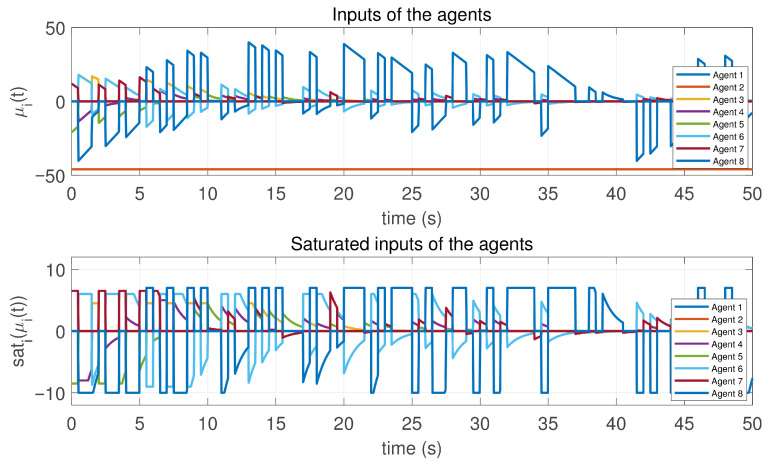
Inputs and saturated inputs of the controlled agents in Case 1 of Example 2.

**Figure 15:**
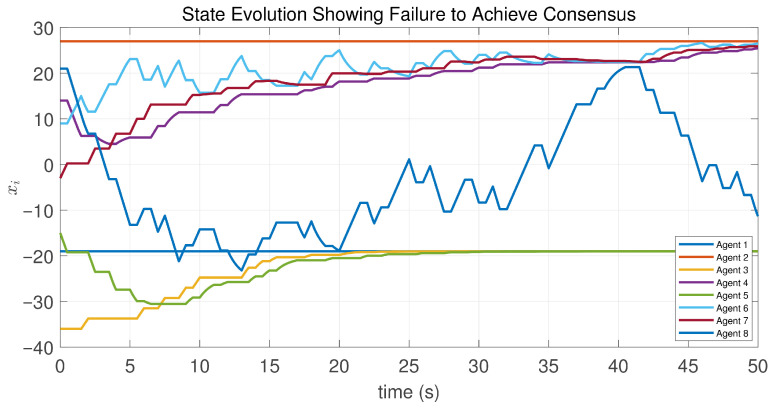
State evolution of the controlled agents in Case 1 of Example 2.

**Figure 16:**
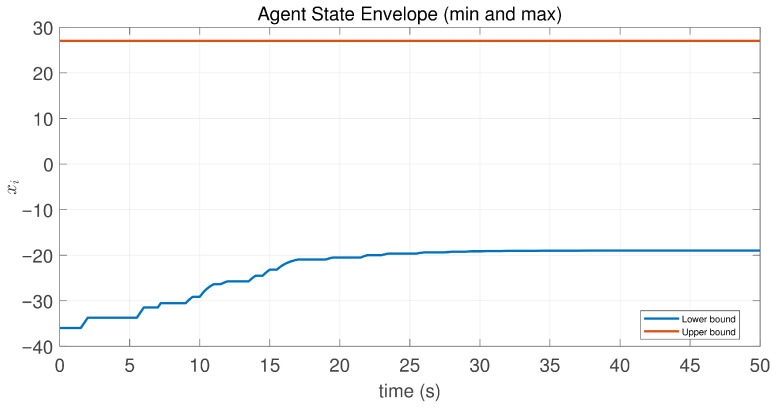
State envelope of the controlled agents in Case 1 of Example 2.

**Figure 17:**
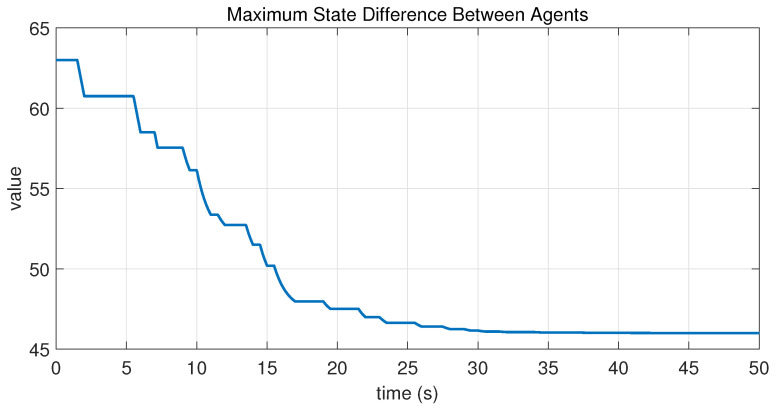
State evolution of $\max_{i,j\in N}\left|x_{i}-x_{j}\right|$ in Case 1 of Example 2.

This case uses the same initial states as Example 1. Note that agent 1 is the only neighbor of agent 2, and the initial state difference $x_{2}\left(0\right)-x_{1}\left(0\right)$ is greater than zero. According to the preceding analysis, this implies that the control input of agent 2 remains constant. Consequently, its saturated control input is zero, and thus, its state remains unchanged, as shown in Figure 14 and Figure 15. Furthermore, Figure 16 and Figure 17 show that the maximum state difference between agents does not converge to zero, which is consistent with Theorem 1.

**Case 2.** In this case, the switching sequence of digraphs $G_{1},G_{2},G_{3}$ follows a cyclic order: $G_{1}\to G_{2}\to G_{3}\to G_{1}\to G_{2}\to G_{3}\to \cdots$. It can be verified that the topology satisfies Assumption 1. For agents i=1,3,4,5,6,7,8, we adopt the same saturation functions as in Case 1 of Example 2, while the saturation function for agent 2 is defined as

(48) $$\begin{array}{c} {\mathrm{sat}}_{2}\left(r\right)=\left\{\begin{array}{cc} -0.001, & \mathrm{for}\;r<0,\\ r, & \mathrm{for}\;0\leq r<4,\\ 4, & \mathrm{for}\;r\geq 4. \end{array}\right. \end{array}$$

**Figure 18:**
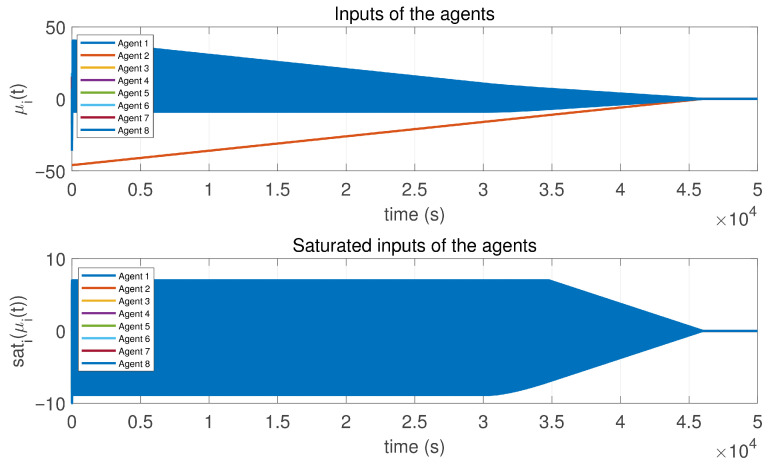
Inputs and saturated inputs of the controlled agents in Case 2 of Example 2.

**Figure 19:**
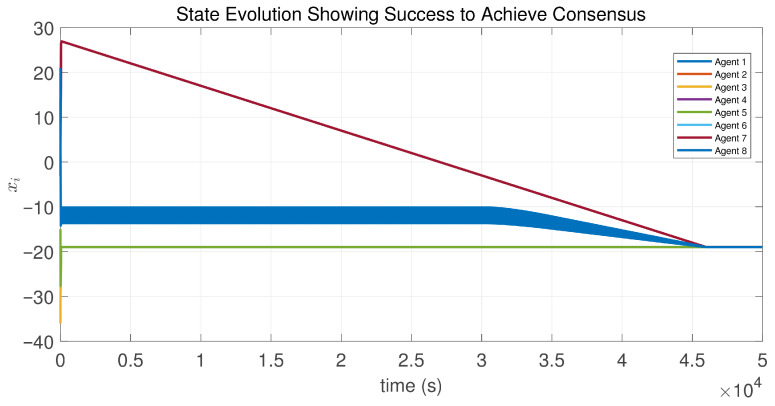
State evolution of the controlled agents in Case 2 of Example 2.

**Figure 20:**
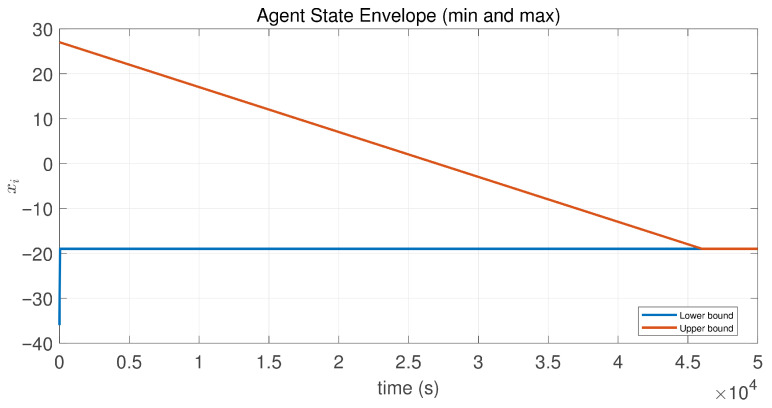
State envelope of the controlled agents in Case 2 of Example 2.

**Figure 21:**
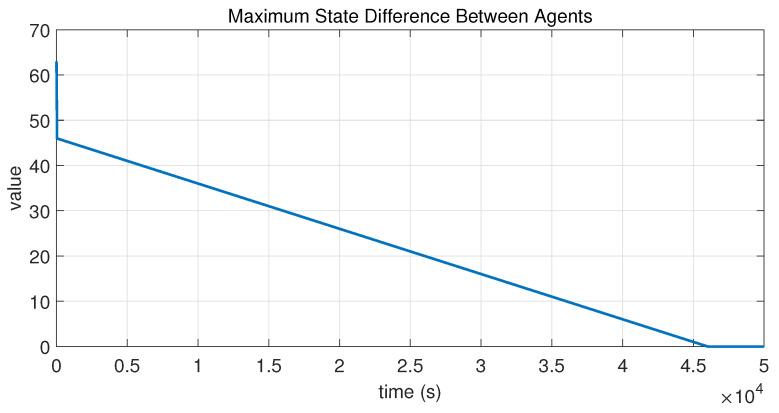
State evolution of $\max_{i,j\in N}\left|x_{i}-x_{j}\right|$ in Case 2 of Example 2.

With the same initial states as in Case 1 of Example 2, the simulation results are shown in Figure 18, Figure 19, Figure 20 and Figure 21. It can be seen that even with a very small but non-zero lower bound $s_{2}^{L}=-0.001$, state convergence can still be observed and consensus can eventually be achieved, despite the very slow convergence speed.

Thus, the two examples above demonstrate that the simulation results are consistent with the theoretical analysis.

**Remark** **4.** *It should be noted that an edge dwell time of $\tau_{D}=0.5$ s was used in both of the above examples. However, the determination of the switching period varies from case to case. In particular, when the switching sequence of the digraphs is fixed, the switching period can be easily determined. In contrast, when the digraphs switch randomly, the length of the switching period depends on the specific sequence generated.*

## 5. Conclusions

This paper solved the consensus control problem of saturated continuous-time multi-agent systems with heterogeneous asymmetric saturation constraints. Specifically, the communication topology was permitted to be directed, time-varying, and disconnected at each time instant, provided that it satisfied a uniformly quasi-strongly connected condition. Moreover, the saturation bounds were not only asymmetric for each agent but also non-identical across the network. By employing tools of the comparison principle and asymptotic stability theory, it was proven that standard distributed consensus protocols were inherently robust to such generic, non-uniform asymmetric saturation constraint.

Building on these theoretical results, several promising directions merit further investigation, including the following: (i) extending the framework to higher-order saturated multi-agent systems with event-triggered communication to reduce bandwidth consumption; (ii) studying the robust consensus problem in the presence of communication noise and time-varying delays; and (iii) exploring practical applications in domains such as intelligent transportation systems and power grids. In addition, inspired by references [44,45], the security of multi-agent systems represents another important avenue for future research.

## Figures and Tables

**Table 1:** Comparison of the developed and some existing results on consensus with saturation constraints.

Results	Graph	Constraint Type I	Constraint Type II
[11]	fixed topology	heterogeneous	asymmetric
[35]	fixed topology	heterogeneous	symmetric
[33]	fixed undirected topology	homogeneous	symmetric
Our Result	directed switching topology	heterogeneous	asymmetric

## Data Availability

Data are contained within the article.

## Abbreviations

The following abbreviations are used in this manuscript:

QSC	Quasi-Strongly Connected
UQSC	Uniformly Quasi-Strongly Connected
Id	Identity Function
N	Vertex Set
E	Edge Set
